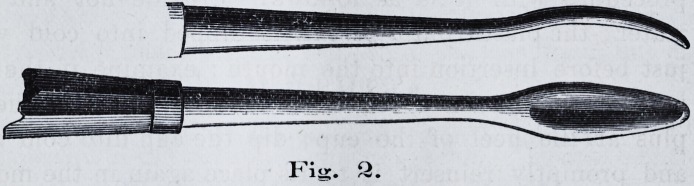# Taking Upper Impressions of the Mouth

**Published:** 1874-08

**Authors:** Geo. S. Fouke

**Affiliations:** Westminster, Md.


### ARTICLE II.

### *Taking Upper Impressions of the Mouth.*

BY GEO. S. FOUKE, WESTMINSTER, MD.

Compression of the alveolar and palatine surfaces by direct manipulation and diversified pressure, after the ordinary perpendicular pressure is made, being of great value and efficacy in performing the operation, the taking of an impression of the roof of the mouth is strictly analogous in a mechanical sense, to the process of ordinary "moulding."

The simple process of "*running the mould*," by which the skilled artisan accomplishes his task of getting a correct impression of the model, is the exact process we have sought to utilize in taking a practically correct impression of the mouth for dental purposes. The solid metal cup used for taking upper impressions being unsuited to this mode or process of manipulation, we devised the cup spoken of in the May number of the *Journal*, and which is here illustrated in the cut, Fig. 1.

Fig. 1.

The cup consists of a strong metallic frame, designed to support the flexible inside lining attached thereto. The combination of the flexible movable floor with the metal frame affords ready means for compressing the entire surfaces of the upper jaw. The pressure is made against the canvas lining C. C., first with the fingers, and finishing the compression with a "ramming" tool suited for the purpose. We use a tool for this purpose as seen in cut, Fig. 2.

Fig. 2.

In the May number of the *Journal* we promised to recur to the subject of taking upper impressions for the purpose of presenting some peculiar features of our process or mode of operation. We will now endeavor to do so briefly.

It may be remarked here that the important and valuable *addendum* of a movable and flexible bottom to the implement we have devised, renders it an easy task to get a correct impression either in wax, gutta-percha, plaster, or any other suitable material. It is not so much the sort of material employed, as it is the manner of working it, that insures the perfection of the work. The perfectly distributed pressure against the cushioned mass of gutta percha, wax, or whatever is used instead, is the essential feature in the process; and when this is skilfully and properly done, impressions of most remarkable correctness are the result.

In taking impressions with the cup we have described, we have recommended the removal of the cup from the mouth, and its re-insertion as often as may be found necessary; except when plaster is used, when of course this would be impossible. In using wax or gutta-percha, the withdrawing of the cup from the mouth is a great relief to the patient, and it will be found advantageous to the operator likewise. He can examine the work and see how the operation is progressing. He has perfect control of the material in the cup, and removing and replacing of the impression will not interfere at all with the final result, but rather help to reach it the more easily and certainly.

In taking a wax impression, the process is very simple, and needs no explanation; but in the use of *gutta percha* it may be well to give some directions. *Gutta percha* is our favorite material for taking impressions, and our method of procedure with it is as follows: Provide hot and cold water; the plastic *gutta percha* is dipped into cold water just before insertion into the mouth; examine if the start is right; if you have too much material pinch off the surplus at the heel of the cup; dip the cup into cold water and promptly reinsert it to its place again in the mouth; after pressing up the cup pretty well, begin pressure against the canvas bottom, and carefully mould the material against the entire surface of the roof; remove again from the mouth, and dip the impression into cold water, quickly return it to the mouth, and by pressure against the sides of the cup, and also by compressing the canvassed mass of *gutta percha*, using the ramming tool; be sure that the material is brought into perfect contact at all points with the whole of the roof of the mouth; carefully remove now and try the impression, that is, try it in the mouth and satisfy yourself that it is correct. It is well to make the plaster cast at once, or just as soon as the impression is completed. Use no oil, as the plaster does not adhere to the gutta percha. As soon as the cast becomes hard the gutta percha is softened by holding the impression in hot water; the cup is pulled off, and the gutta percha removed from the cast.

Due care and accuracy of manipulation observed in taking the impression, the dentist will secure a cast of surpassing correctness. The fit of the dentures when completed will be very superior.

Through the kind and gentlemanly co-operation of Messrs. Snowden & Cowman, as experienced manufacturers of impression cups, we have been enabled to get up new moulds for the improved cups we have designed; and these gentlemen have been entrusted by us with the manufacture and sale of the cup.

The cup is a novelty. Its superiority over the old cups, which for a long series of years have been considered as all that was wanted for the purpose intended, remains to be tested by an intelligent profession. When the mode of operation it embodies is fully mastered, we feel persuaded that the combination of the movable and flexible lining with the cup, as devised, will take its place as a useful and permanent improvement in mechanical dentistry.

## Figures and Tables

**Fig. 1. f1:**
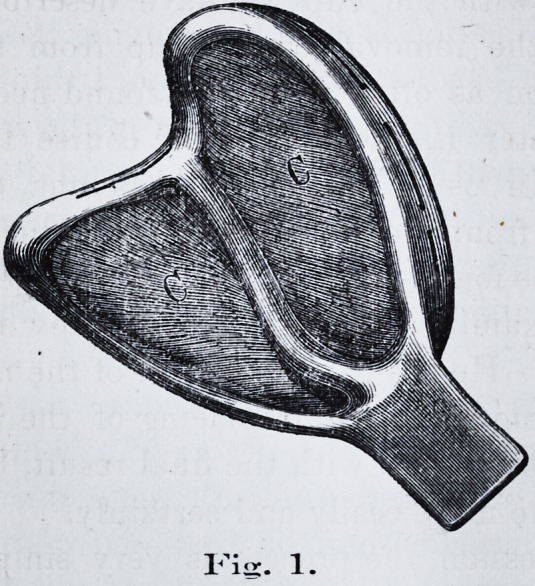


**Fig. 2. f2:**